# Extended Exenatide Administration Enhances Lipid Metabolism and Exacerbates Pancreatic Injury in Mice on a High Fat, High Carbohydrate Diet

**DOI:** 10.1371/journal.pone.0109477

**Published:** 2014-10-07

**Authors:** Rodney Rouse, Leshuai Zhang, Katherine Shea, Hongfei Zhou, Lin Xu, Sharron Stewart, Barry Rosenzweig, Jun Zhang

**Affiliations:** Division of Applied Regulatory Science, Center for Drug Evaluation and Research, U. S. Food and Drug Administration, Silver Spring, Maryland, United States of America; Centro Nacional de Investigaciones Oncológicas (CNIO), Spain

## Abstract

This study expanded upon a previous study in mice reporting a link between exenatide treatment and exocrine pancreatic injury by demonstrating temporal and dose responses and providing an initial mechanistic hypothesis. The design of the present study included varying lengths of exenatide exposure (3, 6 weeks to 12 weeks) at multiple concentrations (3, 10, or 30 µg/kg) with multiple endpoints (histopathology evaluations, immunoassay for cytokines, immunostaining of the pancreas, serum chemistries and measurement of trypsin, amylase, and, lipase, and gene expression profiles). Time- and dose-dependent exocrine pancreatic injury was observed in mice on a high fat diet treated with exenatide. The morphological changes identified in the pancreas involved acinar cell injury and death (autophagy, apoptosis, necrosis, and atrophy), cell adaptations (hypertrophy and hyperplasia), and cell survival (proliferation/regeneration) accompanied by varying degrees of inflammatory response leading to secondary injury in pancreatic blood vessels, ducts, and adipose tissues. Gene expression profiles indicated increased signaling for cell survival and altered lipid metabolism in exenatide treated mice. Immunohistochemistry supported gene expression findings that exenatide caused and/or exacerbated pancreatic injury in a high fat diet environment potentially by further increasing high fat diet exacerbated lipid metabolism and resulting oxidative stress. Further investigation is required to confirm these findings and determine their relevance to human disease.

## Introduction

Drugs active in the Glucagon-Like Peptide-1 (GLP-1) signaling pathway treat type-2 diabetes mellitus. The first of these products, exenatide (EXE), was approved by the FDA in 2005. The on-target effects of GLP-1 pathway drugs have been reported to be efficacious [1]. In spite of null findings for pancreatic injury in initial pre-clinical studies and clinical trial submissions to the FDA, by 2008, adverse event reports indicated a possible link between GLP-1 drugs and acute pancreatitis [2]. This eventually led to labeling changes on GLP-1 drug class products to include a warning for increased risk of pancreatitis. Nevertheless, concerns over adverse events involving drug-induced pancreatitis and/or pancreatic injury with use of these anti-diabetic therapies have persisted rooted in experimental studies [3], [4] as well as adverse event reports. More recently investigators have raised concerns about the role that these drugs may play in the initiation and/or propagation of pancreatic cancer [5].

The interplay of lipid and glucose metabolism in maintaining energy homeostasis has been recognized for years [6]. The influence of altered lipid metabolism on pancreatic β-cell function (insulin response) has also been described [7] and lipotoxicity has been posited as a mechanism for β-cell injury [8] contributing to diabetes. Elevated blood triglycerides are the third most common factor associated with clinical acute pancreatitis [9]. Fatty acids harvested from plasma and incubated with rat acinar cells were able to induce apoptosis and/or necrosis [10] similar to that described in mice fed a high fat diet [11]. In a high fat environment, exenatide altered expression of adipokine genes toward mobilization of fatty acids [12]. Further, exenatide has demonstrated the ability to regulate fatty acid-induced gene expression in both rat and human islet cells [13], [14].

In a previous study, mice developed mild exocrine pancreatic injury when exposed to either a high fat diet (HFD) or EXE and this injury was exacerbated by EXE treatment while on a HFD [11]. However, these results were questioned because only a single dosing concentration was used and a single time point examined with minimal endpoints. Although the authors felt confident in the experimental procedures of this original study, they also agreed that the evidence provided was minimal and provided no hint as to mechanism. The present study expands upon these previous findings by demonstrating dose and time response relationships of drug to injury supported by more endpoints. In the present study, exenatide-induced gene expression in the pancreas was examined for potential mechanisms producing pancreatic injury in association with high dietary fat with a plausible hypothesis emerging.

## Research Design and Methods

### Animals

Male C57BL/6 mice 6 to 8 weeks of age were purchased from Harlan Laboratories (Frederick, MD). Husbandry was consistent with the *Guide for Care and Use of Laboratory Animals, 8^th^ Edition*. Mice were removed from standard chow and placed on a Teklad Custom Research Diet, TD.06415 (Harlan, Frederick, MD), providing 45% of calories through fats (36% saturated, 47% monounsaturated, 17% polyunsaturated), 36% from carbohydrates, and 19% from protein 6 weeks prior to treatment initiation. Health monitoring occurred twice daily with weights taken once weekly. Mice that were moribund or demonstrated signs of malaise, pain, or distress including loss of appetite and lethargy were euthanized. Mice with skin issues were treated with topical antibiotic ointments until the issue resolved. The experimental protocol was approved by the Institutional Animal Care and Use Committee of the White Oak Animal Program located at the White Oak Federal Research in Silver Spring, MD and was conducted in an AAALAC accredited facility at the same location.

### Experimental Design

Three cohorts of eighty mice represented three experimental time points (3, 6, or 12 weeks). Cohorts of eighty were divided into one saline and three EXE (3, 10, or 30 µg/kg) treatment groups of 20 mice each. Mice were fed HFD for six weeks prior to treatment and were then maintained on HFD for 3, 6, or 12 weeks while receiving daily subcutaneous injections of saline or EXE (Creative Peptides, Shirley, NY). Weekly, mice were weighed and dosing adjusted accordingly. At each time point (3, 6, and 12 weeks), 20 mice from each treatment cohort were anesthetized with isoflurane and euthanized by exsanguination twenty-four hours following their final dosing. Blood was collected from each mouse for serum harvest. From each treatment group, 10–12 pancreata were processed for histology evaluation, 5 had RNA extracted, and 3 were preserved at −80°C for future use.

### Pro-inflammatory cytokines and serum chemistries

Pro-inflammatory cytokines were measured in sera using the Meso Scale Discovery platform (Meso Scale Discovery, Gaithersburg, MD) with the Mouse ProInflammatory Multi-Spot 7 Spot Plate (Meso Scale Discovery, Gaithersburg, MD) to determine concentrations of interleukin-1b (IL-1b), interleukin-12p70 (IL-12p70), interferon gamma (IFNγ), interleukin-6 (IL-6), keratinocyte chemoattractant (KC), interleukin-10 (IL-10), tumor necrosis factor alpha (TNFα). Serum concentrations of albumin, total protein, alkaline phosphatase, alanine aminotransferase (ALT), amylase (AMY), total bilirubin, calcium, phosphorus, glucose, sodium, potassium, globulins, blood urea nitrogen, and creatinine were determined on a Vetscan Model 200 Blood Analyzer using Vetscan Comprehensive Diagnostic Profiles (Abaxis, Union City, CA).

### Serum Trypsin, Amylase, Lipase

Trypsin substrate Boc-Gln-Ala-Arg-MCA (Peptides International, Louisville, KY) was purchased and incubated with serum in 48-well plates at 37°C. The plate was read by a fluorometric microplate reader at ex380 nm/em440 nm at 10 minute intervals for one hour. Trypsin activity was reported relative to trypsin standards (Thermo Scientific, Rockford, IL). Amylase and lipase activity was quantified using QuantiChrom α-Amylase and Lipase Assay Kits (BioAssay systems, Hayward, CA), respectively, according to manufacturer’s directions. Total protein was quantified by BCA Protein Assay Reagent (Thermo Scientific, Rockford, IL).

### Histopathology

Pancreatic tissues were fixed in 10% neutral phosphate-buffered formalin, embedded in paraffin, sectioned at a thickness of 5 µm (coronal section through entire pancreas), and stained with hematoxylin-eosin (H&E) for routine histopathology evaluation. Morphological changes in tissue sections were assessed microscopically by a pathologist using a previously published blinded evaluation method [15], [11].

### Immunohistochemical (IHC) Staining and Digital Image Analysis

Immunohistochemical staining was undertaken to quantify injury observed in 30 µg/kg EXE treated mice relative to controls after 12 weeks of treatment. A proliferative marker (Ki-67), in situ detection of apoptosis by TdT-mediated dUTP-biotin nick end labeling (TUNEL), and regenerating islet-derived 3 gamma (Reg3γ), a previously described acinar cell stress marker [16], were used on single sections from all control and 30 µg/kg EXE treated mice. A ScanScope digital scanner (Aperio, Vista, CA) created digital images of slides that were then downloaded into Visiomorph (Visiopharm, Denmark) image analysis software to quantify positively stained nuclei or general staining intensity. From whole slide scans, images (1280×1024 pixels) were selected from each stained slide. Color bands for pixel classification were selected to highlight image features. Ki67 used a DAB color band with Bayesian classification, resulting in an output of the number of Ki76-positive labels in the image. Reg3γ used red, green, blue, and hematoxylin color band input with Bayesian classification to yield the area of positive staining in the image. Apoptosis stains were segmented using the red color band and assigning threshold pixel values to label apoptotic cells, with a total count of positive cells per image. Independently, a pathologist also provided a blinded semi-quantitative evaluation of the staining.

### IHC Staining

Paraffin blocks were sectioned (coronal section through entire pancreas) at 4 µm. Immunohistochemistry was performed on the Discovery XT autostainer (Ventana Medical Systems, Tucson, AZ). After deparaffinization, Tris-EDTA solution, CC1 (Ventana Medical Systems), provided antigen retrieval. Primary antibodies, Reg3γ at 1∶75 (AP5606c; Abgent, San Diego, CA) and Ki-67 at 1∶50 (RM-9106-S; Thermo Scientific, Fremont, CA), were incubated on tissue sections for 1 hour at room temperature. Negative controls were run simultaneously using Rabbit IgG (Sigma, St. Louis, MO) in place of the primary, matched to the protein concentration of the working primary dilution. Secondary antibodies were applied using Vectastain Elite ABC Kit (Vector Laboratories, Burlingame, CA) and detection and visualization were carried out using the DAB Map Detection Kit (Ventana Medical Systems). Tissue sections were counterstained with hematoxylin.

### TUNEL Staining

Paraffin embedded pancreas samples were sectioned at 4 µm and deparaffinized to PBS. Staining was performed using the CardioTACS *In Situ* Apoptosis Detection Kit (Trevigen, Inc., Gaithersburg, MD) according to the manufacturer’s instructions. Briefly, tissue sections were treated with Proteinase K for 15 min followed by 2% H_2_O_2_ for 5 minutes. Sections were incubated with a reaction mixture (TdT, MnCl_2_, and dTNP) at 37°C for 60 minutes, and then with streptavidin-horseradish peroxidase for 10 minutes. In the final step, uncolored soluble chromogen (Trevigen blue label) was enzymatically converted into an insoluble blue-colored complex, precipitating at the site of the reaction following incubation for 3 minutes. These sections were counterstained with Red counterstain C. The DNA fragmentation of apoptotic nuclei was indicated by a blue color [17].

### IHC and TUNEL Semi-Quantitative Scoring

Immunoreactivity of Reg 3γ, TUNEL, and Ki-67 was assessed on the basis of immunostaining intensity and the pattern of Reg 3γ stained cytoplasm and TUNEL or Ki-67 stained nuclei. The results were expressed as a semi-quantitative score: 0 = no positive staining; 1+ = randomly distributed single or a few cells expressing weak staining; 2+ = closely concentrated and clustered cells expressing moderate staining; and 3+ = entire lobular cells expressing intensive staining. A mean was then calculated for the group.

### RNA Extraction and Gene Expression Analysis

Pancreas tissue was resected, preserved in RNALater, and stored at −80 C. After thawing, 2.5 mg of each sample were processed using miRNeasy Mini Kits (Cat #217004) (Qiagen, Valencia, CA). Samples were homogenized in 700 µL of Qiazol Lysis Reagent (Qiagen), for 5 minutes at 50 hz in a TissueLyser LT (Qiagen), and processed using the automated purification of total RNA on a Qiacube (Qiagen) according to “Purification of total RNA, including small RNAs, from animal tissues & cells (aqueous phase), version 2 (April 2010)” standard protocol, miRNeasy Mini Handbook (1073008 07/2012). Mean yield per 2.5 mg of pancreas tissue was approximately 35 µg, determined by NanoDrop spectrophotometer (Thermo Scientific, Wilmington, DE). Average RIN values were approximately 5.4+/−1.4, as assayed by a 2100 Bioanalyzer Instrument (Agilent Technologies, Santa Clara, CA), using an Agilent RNA 6000 Nano Kit (cat #5067–1511). This RIN mean was consistent with our previous experiences in RNA extraction from murine pancreatic tissue.

Expression analysis was performed by Expression Analysis, Inc. (Durham, NC). The 3 samples with the highest RIN from each 12 week treatment group were used to probe treatment effect on gene expression. Total RNA samples (100 ng) were converted into cDNA using Ovation WGA FFPE System (NuGEN, Part No. 6200). Second strand cDNA was purified with Agencourt RNAClean beads and followed by SPIA amplification. Amplified product was purified using Agencourt RNAClean beads and quantitated using a NanoDrop ND-8000 spectrophotometer. Target preparation was performed using 4 µg of amplified cDNA and the NuGEN FL-Ovation cDNA Biotin Module V2. Fragmented and biotin-labeled target was hybridized to Affymetrix GeneChip Mouse Genome 430 2.0 microarrays.

Raw gene expression data were first analyzed using the ArrayTrack bioinformatics tool. Expression data were then imported to Ingenuity Pathway Analysis (IPA; Qaigen, Redwood City, CA; Build version: 261899; Content version: 17199142) tool for further analyses of pathway, biofunction, and toxicity using general and pancreas-specific knowledge bases.

### Statistics

Non-gene expression analyses were completed in SigmaPlot 12 (Systat Software, Inc., San Jose, CA) software using 2-way analysis of variance (2-way ANOVA) with Holm-Sidak *post-hoc* method, or t-test, or corresponding non-parametric tests as appropriate. In each case, significance was conferred at p<0.05. Gene expression data were evaluated with the ArrayTrack bioinformatics tool with a robust multichip average algorithm and quantile normalization. Differentially expressed genes were defined by p<0.05 and fold change >1.3 in Welch t-test comparing treatment and control groups.

## Results

### General toxicity and weight changes

Experimental attrition due to trauma, tumors, or urinary tract obstruction occurred throughout the experimental time course; 5 in the 3-week treatment group (2 from the low and medium dose groups and one from the high dose group); 1 in the 6-week group (1 control mouse); 3 in the 12-week group (1 mouse from each EXE group).

Overt toxicity was not observed in treated mice. High dose EXE (30 µg/kg) mice gained significantly less weight (were less obese) than control mice at each sacrifice point (3, 6, and 12 weeks) with similar non-significant tendencies seen with both lower doses of EXE at all three time points (Table S1). Although in the normal range, ALT was decreased in a dose- and time-dependent relationship to EXE treatment and serum globulin was decreased in high dose EXE mice following both 6 and 12 weeks of treatment (Table S2). There were no significant changes detected in serum lipase. Serum trypsin was diminished in the high dose EXE groups at both 6 and 12 weeks and the medium dose group at 6 weeks (Table S3). Conversely, serum amylase was significantly greater in the high dose EXE groups at 6 and 12 weeks (Table S3). Serum pro-inflammatory cytokines were not increased by EXE (all individual animal data is available in Excel Workbook S1).

### Histopathologic observations

**Figure 1:**
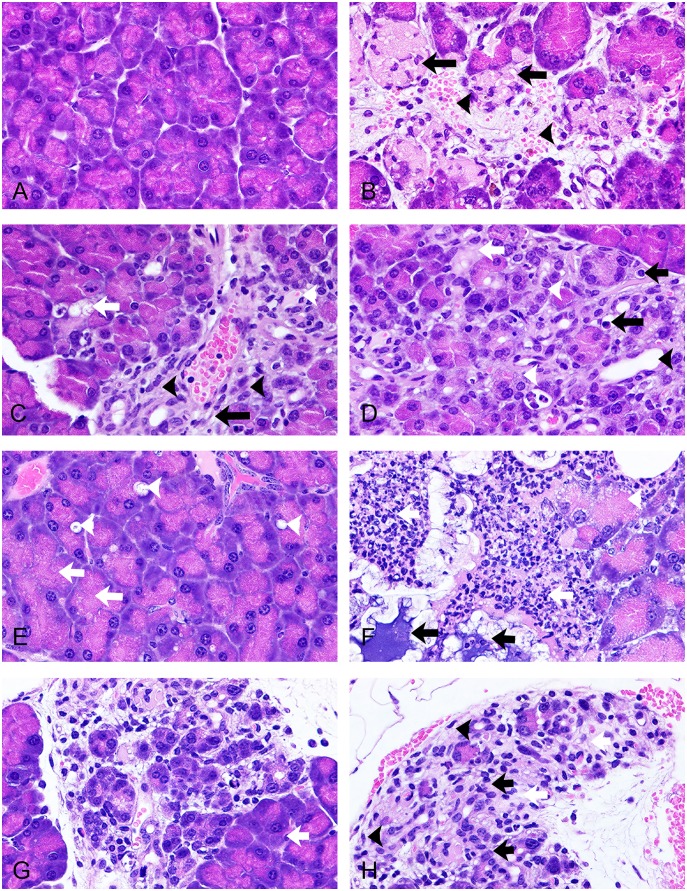
Representative micrographs showing the range of exocrine pancreatic injury associated with drug doses and time points in HFD mice. (A) Saline, 3 weeks; normal acinar cell morphology representative of all control time points. (B) 30 µg/mg exenatide, 3 weeks, acute focal acinar cell necrosis (black arrows), interstitial edema with hemorrhage (black arrowhead). (C) 30 µg/mg exenatide, 6 weeks, periductal acinar cell autophagy (white arrow), early ductal hyperplasia (black arrowhead), capillary dilatation (white arrowhead), and increase in early fibrosis (black arrow). (D) 30 µg/mg exenatide, 6 weeks, acinar cell autophagy, apoptosis (black arrows), ductal dilatation (black arrowhead) and early ductal hyperplasia (white arrowheads) with deposit of protein-rich materials in the interstitium (white arrow). (E) 3 µg/mg exenatide, 12 weeks, acinar cell hypertrophy (increased ratio of zymogen granules; white arrows) and autophagy (white arrowheads). (F) 3 µg/mg exenatide, 12 weeks, severe acinar cell necrosis (white arrowhead), interstitial inflammation (white arrows), and fat necrosis with calcification (black arrows). (G) 3 µg/mg exenatide, 12 weeks, severe, complex pancreatic injury including acinar cell loss, interstitial edema, and early ductal hyperplasia and fibrosis. Note adjacent non-affected (white arrows) acini. (H) 3 µg/mg exenatide, 12 weeks, severe acinar cell loss, atrophy, ductal hyperplasia (black arrows), fibroblast proliferation (black arrowheads), and deposit of protein-rich materials in the interstitium (white arrows). A–H, X630, H&E stain.

**Figure 2:**
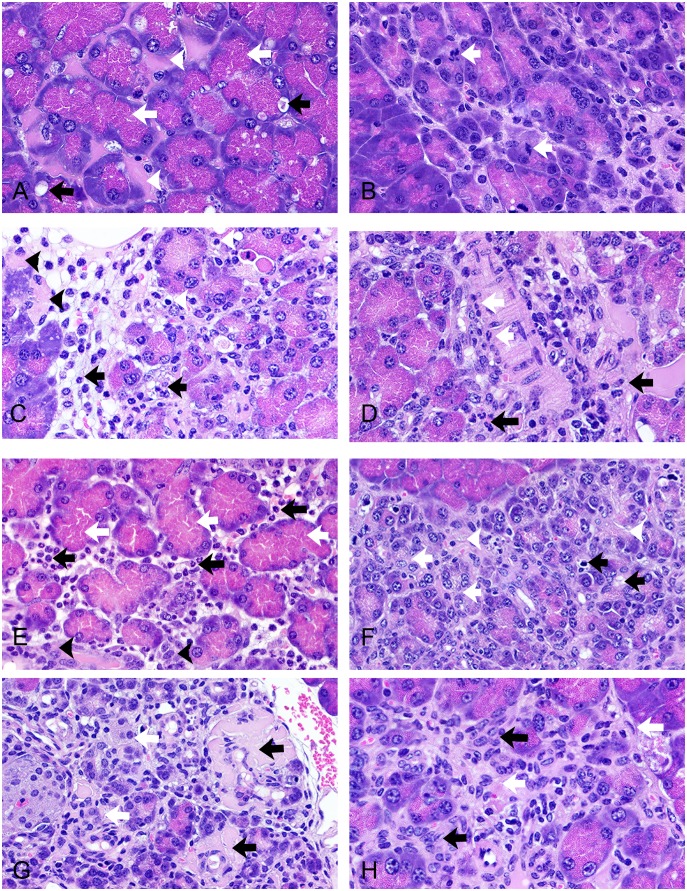
Representative micrographs showing range of exocrine pancreatic injury associated with drug doses and time points in HFD mice. (A) 10 µg/mg exenatide, 12 weeks, acinar cell hypertrophy (white arrows) in association with autophagy (black arrows), and interacinar edema (white arrowheads). (B) 10 µg/mg exenatide, 12 weeks, several mitoses (white arrows) indicative of cell proliferation (hyperplasia). (C) 10 µg/mg exenatide, 12 weeks, acinar cell apoptosis (white arrowheads), necrosis (black arrowheads) and inflammatory edema (black arrows) in the interstitium. (D) 10 µg/mg exenatide, 12 weeks, vasculitis (white arrows) in association with interstitial inflammation and edema (black arrows). (E) 30 µg/mg exenatide, 12 weeks, severe interstitial inflammation and edema (black arrows) in association with acinar cell hypertrophy (white arrows) and ductal hyperplasia (black arrowheads). (F) 30 µg/mg exenatide, 12 weeks, the most complex pancreatic injury: mixture of a variety of acinar cell injuries (black arrows), ductal hyperplasia (white arrows), interacinar fibrosis (white arrowheads). (G) 30 µg/mg exenatide, 12 weeks, deposit of a protein-rich fluid (exudate) surrounding injured arterioles (black arrows) and ductal hyperplasia (white arrows). (H) 30 µg/mg exenatide, 12 weeks, increase in fibroblast proliferation (interstitial fibrosis; white arrows) and ductal hyperplasia (black arrows). A–H, X630, H&E stain.

Table 1 presents pancreatic lesion scores in mice on HFD treated with saline or EXE (3, 10, 30 µg/mg, once daily by subcutaneous injection) for 3, 6, or 12 weeks and demonstrates a significant dose- and time-dependent increase in EXE-associated exocrine pancreatic injury. Figure 1 and Figure 2 contain representative images of EXE associated injury to the exocrine pancreas relative to EXE dose and time exposure. The lesions seen in EXE treated mice on HFD were similar to those seen in saline treated mice on HFD but were more prevalent and more severe across doses and time as reflected in Table 1. Although low levels of acinar cell hypertrophy, autophagy, and apoptosis were observed, the histology of the exocrine pancreas in control animals was comparable over periods of 3, 6, and 12 weeks of saline treatment and showed largely normal histology (Figure 1A). EXE for 3 weeks at even the high dose did not yield sufficiently uniform injury to produce significant group scoring differences between control and EXE treated mice. However, EXE treated groups did have consistently larger mean scores and individual animals within each EXE dose group demonstrated acinar cell injury and early vascular injury and fat necrosis. Focal acinar cell necrosis and interstitial inflammation and edema (Figure 1B) were identified in some high dose mice after 3 weeks of EXE. EXE for 6 weeks produced dose-dependent pancreatic injury. Foci of acinar cell autophagy, apoptosis, and necrosis as well as ductal hyperplasia increased with EXE exposure concentrations being most apparent in high dose mice (Figures 1C, 1D).

**Table 1:** Histopathology evaluations link time exposure and dose concentration dependent increases in exocrine pancreatic injury to exenatide treatments.

Categories of individualinjury	Mean Category and Cumulative Histopathology Score of Injury in Exenatide Treated Mice(0–3 range for each category)											
	3 weeks, EXE				6 weeks, EXE				12 weeks, EXE			
	0 µg(n = 12)	3 µg(n = 10)	10 µg(n = 10)	30 µg(n = 10)	0 µg(n = 10)	3 µg(n = 11)	10 µg(n = 12)	30 µg(n = 12)	0 µg(n = 12)	3 µg(n = 11)	10 µg(n = 11)	30 µg(n = 12)
Acinar cell hypertrophy	0.7	0.6	1.3	1.1	0.7	1.2	1.3	1.6	0.6	1.9	2.0	2.6
Acinar cell autophagy	0.8	0.9	1.2	1.1	0.9	1.4	1.4	2.0	0.6	1.6	1.6	2.5
Acinar cell apoptosis	1.1	1.0	1.1	1.1	1.0	1.6	1.5	2.1	1.1	2.0	2.1	2.5
Acinar cell necrosis	0.0	0.1	0.1	0.4	0.0	0.1	0.3	0.8	0.0	0.5	0.6	1.8
Vascularinjury	0.0	0.3	0.0	0.0	0.1	0.1	0.2	0.1	0.0	0.1	0.4	1.6
Interstitial inflammatory edema	0.9	0.9	1.2	1.0	0.6	0.2	1.0	0.8	0.8	0.3	2.5	2.8
Fat necrosis	0.0	0.1	0.0	0.0	0.0	0.1	0.2	0.0	0.0	0.3	0.3	2.5
Duct changes	0.3	0.4	0.6	0.6	0.7	1.0	1.3	1.5	0.5	1.8	2.0	2.8
Acinar cell atrophy	0.2	0.8	0.5	0.6	0.2	1.2	1.9	2.7	0.5	2.5	2.8	2.9
**Group Mean Scores of** **combined** **all Injuries**	4.0	5.3	6.0	5.9	4.2	6.9*	8.8*	11.0*	4.0	12.8*	14.5*	18.6*

µg = microgram per kg dose of exenatide for treatment group; weeks = the weeks of daily subcutaneous exenatide injections; *indicates significant difference (p<0.05) from control.

EXE for 12 weeks resulted in the most consistent and striking dose-dependent pancreatic lesions ranging in low dose (3 µg/mg) mice from frequent acinar cell autophagy (Figure 1E) to severe fat necrosis (Figure 1F) with detectable acinar cell atrophy, ductal hyperplasia, and early fibrosis (Figure 1G, 1H). After 12 weeks of EXE, changes in the mid dose (10 µg/mg) group ranged from more frequently observed autophagy (Figure 2A) and mitosis suggestive of proliferation (Figure 2B) to interstitial fibrosis characterized by proliferating fibroblasts intertwined with injured acinar cells (Figure 2C) and ductal hyperplasia associated with perivascular inflammatory cells (Figure 2D). Following 12 weeks of EXE, changes in the high dose mice ranged from those changes described for the two other dose groups to severe acute inflammation and acinar cell hypertrophy (Figure 2E) and more severe, complex, and comprehensive injury to the exocrine pancreas including an intermixture of injured acinar cells with ductal hyperplasia (Figure 2F), the intermingling of atrophied acinar cells, hyperplastic ducts, and injured aterioles with extravasation of fibrinoid material (Figure 2G), and combined interstitial fibrosis and hyperplasia of the pancreatic ducts (Figure 2H).

### Immunohistochemical studies

**Figure 3:**
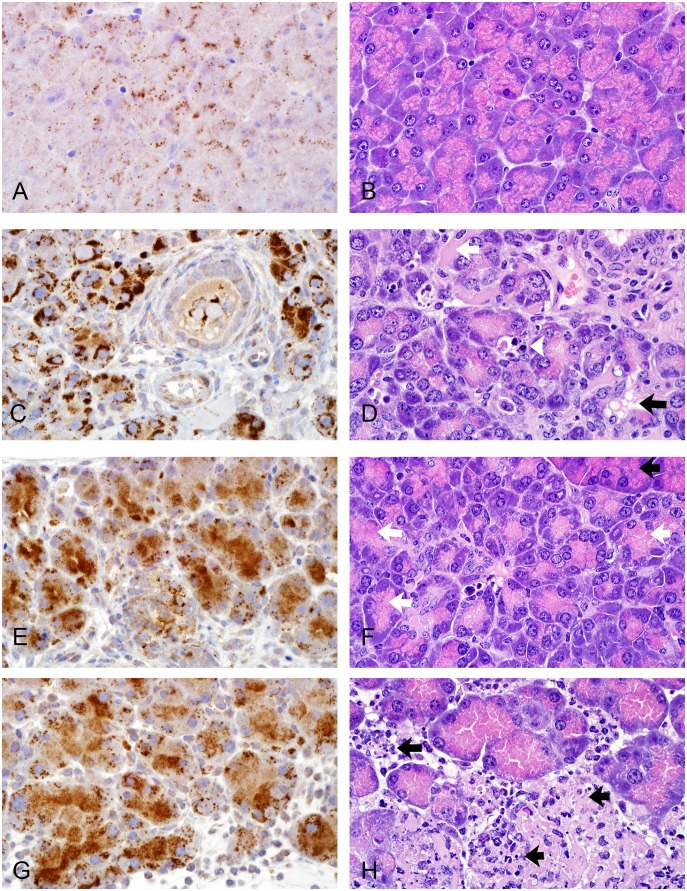
Representative micrographs comparing Reg3γ immunoreactivity to morphologic change in HFD mice. (A & B) Saline, 12 weeks, weak Reg3γ immunoreactivity (A) characterized by discrete fine punctate and sparse coarse particles in the cytoplasm of acinar cells observed in areas of normal acinar cell morphology (B). (C & D) 30 µg/mg exenatide, 12 weeks, increased Reg3γ immunoreactivity (C) characterized by strongly intensive staining occupying almost all cytoplasm of acinar cells seen in areas demonstrating severe acinar cell injuries (D) including autophagy, apoptosis, necrosis. (E & F) 30 µg/mg exenatide, 12 weeks, increased Reg3γ immunoreactivity (E) characterized by intensive homogenous staining predominantly located in the zymogen granular zones of acinar cells. The staining pattern matched areas of acinar cell hypertrophy (F). Note normal acinar cells of separate lobule in top right corner. (G & H) 30 µg/mg exenatide, 12 weeks, Reg3γ showed a staining pattern (G) with distinct coarse particles in non-zymogen granular zones in areas of inflammation and necrosis (H). A–H, X600, A, C, E, G (IHC for Reg 3γ); B, D, F, H (H&E stain).

**Figure 4:**
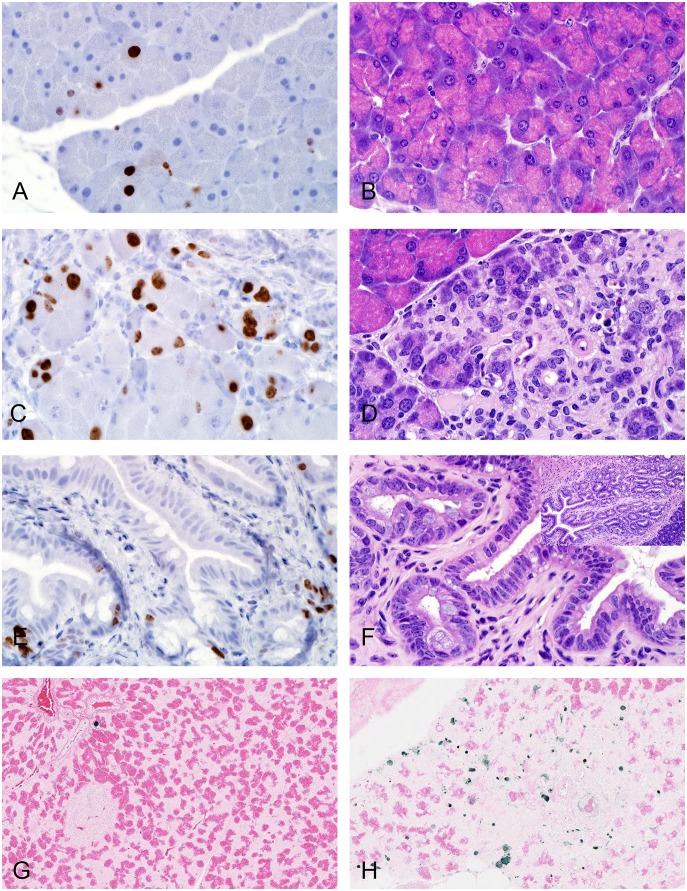
Representative micrographs comparing proliferative (Ki-67) and apoptosis (TUNEL) immunoreactivity to morphologic changes in HFD mice. (A & B) Saline, 12 weeks, Ki-67 staining (A) was occasionally identified in nuclei in areas of normal acinar and centroacinar cells (B). (C & D) 30 µg/mg exenatide, 12 weeks, increased Ki-67 immunoreactivity (C) characterized by more frequently stained nuclei in acinar, centroacinar, ductal, and interstitial cells observed in areas of severe acinar cell injury (D) including autophagy, apoptosis, and necrosis and ductal metaplasia. Two nuclei placed side by side with basophilic stained cytoplasm are suggestive of cell proliferation (hyperplasia). (E & F) 30 µg/mg exenatide, 12 weeks, increased Ki-67 immunoreactivity (E) characterized by nuclear staining in epithelial cells of the main duct observed in areas of pseudostratified columnar epithelium with increased number of goblet cells (F). Inset in F showing main pancreatic ductal cell proliferation. (G) Saline, 12 weeks, TUNEL staining revealed an occasional acinar cell undergoing apoptosis. (H) 30 µg/mg exenatide, 12 weeks, revealed many apoptotic cells in areas associated with acinar cell injury. A–H, X600, A, C, E, (IHC for Ki-67); B, D, F (H&E stain); G & H (TUNEL). Inset in F, X200.

Immunostaining for Reg3γ, Ki-67, and apoptosis in mice treated with 30 µg/kg EXE for 12 weeks was considerably greater in injured areas in EXE treated mice compared to normal areas of morphology in controls (Table 2). An independent semi-quantitative analysis of this staining confirmed these findings (Table 3). Low Reg3γ immunoreactivity correlated with normal acinar cells in control mice (Figures 3A, 3B). High Reg3γ immunoreactivity was associated with areas of acinar cell hypertrophy, autophagy, apoptosis, and necrosis (Figures 3C–3H) seen in EXE treated mice. Low Ki-67 immunoreactivity correlated with normal acinar cells and centroacinar cells (Figure 4A, 4B) in controls. High Ki-67 immunoreactivity was associated with injury of acinar, centroacinar, small ductal cells, and interstitial cells (Figures 4C–4D) in EXE treated mice. Epithelial cells of main pancreatic ducts that strongly stained for Ki-67 were usually located in pancreatico-duodenal junctions (Figure 4E), sometimes appearing as pseudostratified columnar cells (Figure 4F). Goblet cells of these ducts were somewhat enlarged and appeared to be proliferating in a few foci. In sections labeled with TUNEL, a single positive nucleus (Figure 4G) was occasionally found in acinar cell areas with normal H & E morphology (controls) while numerous positive nuclei and fragmented nuclei were located in severely injured areas (Figure 4H) in EXE treated mice.

**Table 2:** Group mean of digital analysis of immunohistochemical staining reveals increased apoptosis, proliferation, and acinar cell stress in areas of exenatide injury.

12 weeks treatment	TUNEL^1^	Ki-67^1^	Reg3γ^2^
**Saline**	3.0±1.0	11.0±6.0	2227±1501
**High dose exenatide**	10.0±5.0*	50.0±10.0*	9858±2985*

1 = expressed in cells per millimeter squared; 2 = expressed in positive pixels per millimeter squared; * = significant at p<0.01; staining described in methods.

**Table 3:** Immunoreactivity scores of Reg 3γ, Ki-67, and apoptosis in pancreas of mice treated with saline or exenatide (30 µg/mg, s.c. injection) for 12 weeks.

Treatment	Reg 3γ immunoreactivity scores				
	0	1+	2++	3+++	Mean score
Saline, 12 weeks (n = 12)	n = 5	n = 6	n = 1	n = 0	**0.6+**
Exenatide 30 µg/mg, 12 weeks (n = 12)	n = 0	n = 2	n = 3	n = 7	**2.4+** *
	**Ki-67 immunoreactivity scores**				
Saline, 12 weeks (n = 12)	n = 5	n = 5	n = 2	n = 0	**0.75+**
Exenatide 30 µg/mg, 12 weeks (n = 12)	n = 0	n = 1	n = 3	n = 8	**2.5+** *
	**Apoptosis immunoreactivity scores**				
Saline, 12 weeks (n = 12)	n = 3	n = 8	n = 1	n = 0	**0.8+**
Exenatide 30 µg/mg, 12 weeks (n = 12)	n = 0	n = 3	n = 4	n = 5	**2.2+** *

*Indicates statistically significant difference from saline (p<0.05).

### Gene expression

Expression analysis revealed EXE related perturbations in genes linked to stem cellular survival and differentiation, and lipid metabolism. Table 4 and Table 5 summarize the expression alterations in functions and genes of mice exposed to 30 µg/kg EXE once daily for 12 weeks relative to saline controls. Although, other time points were not evaluated, stem cell survival (Table 4) and lipid metabolism (Table 5) were the top molecular and cellular functions altered by EXE treatment in all 3 dosing groups after 12 weeks (details for each of these analyses are available in PDF files S1, S2, S3). Strongest signals were found in the high dose group. Decreased stem cell death (Z-score −2.397) was predicted in IPA from directional consistency in 6 of 7 genes (1 undetermined) and an overlap p-value of 0.0104. Further literature review did not clarify the potential role of the undetermined gene in the cell survival/death analysis. Manual literature review was consistent with the IPA analysis and also suggested a broader role of the identified genes in general cell survival, proliferation, and in the case of stem cells decreased differentiation (Table 4 PMID references). IPA predicted increased oxidation of fatty acids (Z-score 2.833) based on directional consistency in 10 of 15 identified genes and overlap p-value of 0.0008. Of the remaining 5 genes, the direction of 4 was undetermined and 1 was determined to have an opposing effect in IPA. A manual literature review supported the IPA predicted increase in oxidation of fatty acids and suggested that the four undetermined genes were also likely to contribute to increased fatty acid metabolism and an environment of oxidative stress (Table 5 PMID references). The single gene predicted to decrease fatty acid oxidation was identified as instrumental in providing an alternate path for fatty acid utilization by increasing glycerolipid synthesis, a process that would not be unexpected in the HFD environment.

**Table 4:** Cell death/survival/proliferation/differentation related gene expression in mice treated with EXE for 12 weeks indicates increased cell survival compared to controls.

Gene		Expression Change		Protein Function	Predicted Impact	Reference PMID
Symbol	Name	FoldChange	p-value			
BRCA1	breast cancer 1, earlyonset	1.58	0.021	DNA repair; oxidative DNA damagerepair	enhanced cell survival	9703501, 23271346, 24314328
AGO4	argonaute RISC catalytic component4	1.615	0.026	Component of RNA gene silencingcomplex; stem cell proliferation andsurvival	enhanced cell survival	19174539, 19393748, 22863743
NRP1	neuropilin 1	2.51	0.034	Co-receptor for growth factors; cellgrowth, proliferation, and survival	enhanced cell survival	15572379, 19337405, 21828096, 22948112
PIK3R1	phosphatidylinositol3-kinase regulatory subunit alpha	1.748	0.029	Cell growth, proliferation, and survival	enhanced cell survival	11784871, 12435753, 21649900, 23810379
POLK	DNA polymerase kappa	1.324	0.047	Facilitates DNA synthesis acrossareas of DNA damage	enhanced cell survival	12432099, 16308320, 16738701, 22487424
HES1	hairy and enhancerof split 1	1.432	0.011	Cell proliferation, survival, anddifferentiation; adult centroacinarcells	enhanced cell survival	10627606, 21205788, 22649105, 23274689
FGFR2	fibroblast growth factor receptor 2	–1.333	0.024	Growth factor receptor; cell growth,proliferation, and survival; highexpression associated with increasedpancreatic cancer risk	undetermined	10339576, 17314281, 22440254, 23136392,

Genes identified in expression studies by Ingenuity Pathway Analysis (IPA) were further investigated via literature review for function of their protein, predicted impact of the expression difference specific to this study, and references consistent with consensus on identified protein function and the predicted impact in this study. When the impact was not predictable via IPA, the impact is listed as undetermined. No protein studies were completed to verify gene expression changes resulted in protein expression changes. p-value indicates the significance of fold-change comparing mean and standard deviation from treated and control mice at a 0.05 cutoff. PMID is the PubMed ID number that will yield the relevant reference; n = 3 per group.

**Table 5:** Oxidation/β-oxidation of fatty acid related gene expression in mice treated with EXE for 12 weeks indicates altered lipid metabolism compared to controls.

Gene		Expression Change		Protein Function	Predicted Impact	Reference PMID
Symbol	Name	Fold Change	p-value			
CPT1A	carnitine palmitoyl-transferase A1	1.706	0.012	FA oxidation; mitochondrial	increased FAbreakdown; oxidativestressresponse	11988095, 15105415, 19302064, 19429947
SIRT1	sirtulin 1	1.621	0.003	FA oxidation; mitochondrial	increased FAbreakdown; oxidativestressresponse	16154098, 19962456, 22497970
NR4A2	nuclear orphan receptor 4A2	1.337	0.035	FA oxidation; mitochondrial	increased FAbreakdown; oxidativestress response	17038671, 20566846, 21757690, 23283970
PPARGC1A	peroxisome proliferator-activated receptor gammacoactivator 1 alpha	2.228	0.041	Lipid metabolism	increased FAbreakdown; oxidativestressresponse	10669761, 12606537, 18079123, 22939990
IRS2	insulin receptor substrate 2	1.536	0.047	Insulin metabolism andbeta oxidation of FA	increased FAbreakdown; oxidativestressresponse	9753293, 16814735, 21829658, 23639858
ACOX1	peroxisomal acyl-coenzymeA oxidase 1	2.36	0.006	FA oxidation; mitochondrial	increased FAbreakdown;oxidative stressresponse	8725145, 8798738, 22521832, 23349482
ADH1C	alcohol dehydrogenase 1C	1.304	0.04	metabolism of alcohol,hydroxysteroid, andlipidperoxidation products	oxidativestress response	16081420, 19382905, 22117533
IGF1R	insulin-like growth factor1 receptor	1.332	>0.001	Beta oxidation of FA;mitochondrial	increased FAbreakdown;oxidative stressresponse	15231693, 17447016, 21907144, 23219871
MLYCD	malonyl Co-A decarboxylase	1.359	0.026	FA catabolism	increased FAbreakdown;oxidative stressresponse	15254578, 18314420, 23211317
LPIN1	Lipin-1	2.342	0.009	Regulate FA oxidation	increased FAbreakdown;oxidative stress response	16950137, 22055193, 23291236
GPAM	glycerol-3-phosphateacyltransferase 1,mitochondial	1.654	0.041	Synthesis of glycerolipids	decrease FAbreakdown;oxidative stressresponse	15102885, 15598672, 16507761, 23908354
BDH2	3-hydroxy-butyratedehydrogenase type 2	1.652	0.04	Ketone metabolism	Undetermined*	16380372, 23941109
PNPLA2	patatin-like phospholipasecontaining domain 2	1.329	0.034	Triglyceride hydrolysis tofree FA and FA oxidation	Undetermined*	18337240, 22158969, 22951290
ECI2	enoyl-Co A delta isomerase2	1.912	0.044	Beta oxidation of FA;mitochondrial	Undetermined*	8486162, 10585880, 11781327, 19866242
HADHA	hydroxyacyl-CoA dehydrogenase/3-ketoacyl-CoA thiolase/enoyl-CoAhydratase, alpha subunit	1.405	0.026	Beta oxidation of FA;mitochondrial	Undetermined*	11390422, 22325456, 24362249

Genes identified in expression studies by Ingenuity Pathway Analysis (IPA) were further investigated via literature review for function of their protein, predicted impact of the expression difference specific to this study, and references consistent with consensus on identified protein function and the predicted impact in this study. When the impact was not predictable via IPA, the impact is listed as undetermined. *Designates undetermined cases in Ingenuity that subsequent literature review suggested would result in increased fatty acid (FA) metabolism and oxidative stress. No protein studies were completed to verify gene expression changes resulted in protein expression changes. p-value indicates the significance of fold-change comparing mean and standard deviation from treated and control mice at a 0.05 cutoff. PMID is the PubMed ID number that will yield the relevant reference; n = 3 per group.

## Discussion

Although still well within expected normal ranges, mean body weight, ALT, and globulin were decreased in EXE treated mice. These decreases were likely related to EXE effects on glucose and lipid metabolism and satiety. Decreases in liver enzymes have been reported in diabetic patients on long term exenatide therapy [18]. There was a time-dependent increase in levels of serum lipase and pro-inflammatory cytokines in all experimental groups that was attributed to increasing exposure to HFD (data not shown). EXE-treated mice demonstrated lower serum trypsin and higher amylase levels. The reason for the decrease in serum trypsin was not identified. Tissue trypsin levels were not measured and the relation between serum and tissue levels was not defined. Amylase increases noted, though significant, were not of a magnitude diagnostic of acute pancreatitis. Serum chemistry alterations typical of acute pancreatitis were not observed in this study. Diffuse acute pancreatitis was not the described histopathology.

Pathology findings in the present study were in general agreement with Rouse et al., 2014 [11]. HFD mice treated with EXE exhibited a dose- and time-dependent increase in EXE-related exocrine pancreatic injury. In addition to early morphological changes (acinar cell hypertrophy, autophagy, and mild apoptosis) as described in experimental pancreatitis models [15], multifocal changes including acinar cell necrosis, vascular injury, and fat necrosis were observed with higher, prolonged EXE doses. Acinar cell atrophy and pancreatic fibrosis (interacinar fibrosis, interlobular fibrosis, coalescing areas of fibrosis) were observed and proliferative and atrophic changes were noted focally in small intercalated ducts with longer term higher doses of EXE. Atrophic changes included proliferative centroacinar cells associated with altered intralobular and main ducts. Similar centroacinar cell proliferation has been proposed as the source of ductal metaplasia [19] that is debated as a precursor to malignant transformation.

Reg3 proteins (α, β, γ) have been described as a family of pancreatic stress proteins with Reg3β and Reg3γ increased in caerulein-induced pancreatic injury [16] characterized by extensive autophagy. Reg3β, also known as pancreatitis associated protein (PAP), and Reg3α have been increased in diabetic mice treated with EXE [20]. Mice treated with EXE after partial pancreatectomy also exhibited a substantial increase in Reg3β [21]. Reg3γ appears to reflect cellular response to injury and play a role in the regeneration of pancreatic β-cells [22], [21] and may have a similar part in acinar cell regeneration. Some reports suggest that Reg3γ immunoreactivity in EXE-treated mice reflects a cellular response to oxidative stress. In a BALB/c mouse model treated with EXE, chromatin immunoprecipitation analyses confirmed Reg3γ as a novel transcription target of Foxa2 (hepatocyte nuclear factor 3β, HNF3β) during pancreatic regeneration after partial pancreatectomy [21]. Previously, Foxa2 was identified as a major upstream regulator of *pdx-1* transcription and expression [23]–[26]. *Pdx-1* is a homeobox gene essential for pancreatic development [26] and maintenance of islet β-cells function. The ability of GLP-1 to activate *pdx-1* expression may be mediated in part by Foxa2 [27]. The present study supports a similar role for Reg3γ in acinar cells.

Increased positive Ki-67 immunoreactivity indicated a cell proliferation effect for EXE. In the Kras ^G12D^ mouse model, EXE increased the number of Ki-67 positive cells in areas of ductal proliferation implying a role for EXE in focal proliferation of the exocrine pancreas and possibly pre-neoplastic PanIn lesion development [28]. Recently, increased pancreatic weights and Ki-67 nuclear staining were linked to prolonged GLP-1 based therapy in humans resulting in unintended pancreatic cell proliferation [5]. Similar pancreatic weight findings and pancreatic cell proliferation in response to GLP-1 based therapeutics had been reported in diabetic mice [20]. Consistent with this human data, the present study generated non-clinical evidence of different types of exocrine pancreatic cells (acinar, centroacinar, ductal cells, and even interstitial cells) undergoing proliferation, however, no neoplastic transformation was identified. In the human study, Ki-67 immunoreactivity was associated with pancreatic intraepithelial neoplasms (PanIN) with exacerbated mucin content within these proliferated cells [5]. In the present study, Ki-67 stained epithelial cell proliferation was observed in main ducts accompanied by expansion of associated mucin-rich goblet cells. These epithelial cells presented as tall columnar cells or pseudostratified epithelial cells in affected areas without morphological evidence of PanIN-like lesions, even though these changes were very similar to illustrations from human studies [29], [30], [5]. The mice used in this study were relatively young with no demonstrated predisposition toward development of PanIN-like lesions. Repetition of this study in mutant Kras mice that develop these lesions may better address the relationship of EXE and pancreatic cancer.

Gene expression changes attributed to EXE treatment largely fell into two pathways: 1) increased lipid metabolism; 2) enhanced stem cell maintenance, survival, and proliferation with diminished differentiation. Experimentally, 12 weeks of exenatide treatment up-regulated genes of lipolytic proteins [31]. Exenatide has enhanced pancreatic lipid metabolism in a high fat environment in vitro and in vivo [12], [20], [31]. Stem cell survival is consistent with findings in β-cells where pro-survival and proliferative gene expression changes have been attributed to EXE treatment through control of Sirt-1 (also differentially expressed in the present study) and other genes that control epigenetic modifications necessary for transcription [13], [14], [32]. EXE has been associated with increased β-cell survival in vitro and ex vivo [33], [34], [35]. In this same mouse model system, another GLP-1 agonist, liraglutide, improved insulin sensitivity while reducing β-cell mass but caused acinar cell proliferation [36]. The in vivo RNA source of the present study was over 90% acinar cells further supporting EXE-related proliferative effects on acinar cells. Intuitively, HFD would be expected to increase lipid metabolism but all of the animals in this study were on HFD with significant increases in lipid metabolism and pancreatic injury identified in EXE-treated mice. EXE exposure in HFD mice has previously been associated with increased fatty acid oxidation in the liver [37], [26]. Increased fatty acid oxidation and β-oxidation found in EXE-treated mice in this study, might be anticipated metabolic responses to deal with the tremendous increase in lipid intake. However, these responses can also be associated with increased oxidative stress, inflammation, and in specific circumstances cancer evolution [38]. Reactive oxygen and nitrogen species have described roles in acinar cell homeostasis and injury progression [39] and oxidative stress has been implicated in the early stages of acute pancreatitis [40], [41], [42], [43]. The enhancement of lipid metabolism perturbation by EXE in the face of increased fat intake may be a mechanism through which EXE initiates or exacerbates pancreatic injury via increased oxidative stress. Given the link between obesity and type-2 diabetes mellitus (the indication for EXE) and the high fat consumption seen is segments of the diabetic patient population, further investigation is merited to define EXE’s influence on fatty acid oxidation and oxidative stress in the pancreas.

Previous research on GLP-1 based therapeutics in general, and EXE specifically, has yielded controversial and contradictory conclusions. The sources of variance impacting study outcomes are not well understood and are no doubt numerous. Unpublished work associated with this study, as well as our previous study [11], implicates several factors. First, Sprague-Dawley rats treated with EXE for 6 weeks had none of the changes previously described by Nachnani et al., 2010 [4]. In addition, changes in the present study were time- or cumulative exposure-dependent. Therefore, both concentration and duration of exposure may have to exceed minimal thresholds to yield injury. Second, consistent with previous reports, EXE-treated Zucker Diabetic Fatty rats on HFD or standard diet demonstrated no discernable differences in pancreatic injury linked to EXE treatment. Further, the differences between mice and rats within the same acute pancreatitis model systems [44], suggest basic physiological differences that are either more protective of EXE injury in rats or that create sufficient variability in rats to make smaller impacts less detectable. Therefore, mice might be more sensitive for detecting EXE related injury. Third, as posited here and previously [11], the sensitivity of mice to EXE may be exacerbated with a HFD. This suggests that the metabolic status of the animals may be a significant source in response variability. We have not conducted similar HFD studies in rats to assess whether exacerbation would be seen in that species as well. Collectively, studies on GLP-1 active therapeutics indicate a complex interaction of risk factors, environment, drug, and possibly disease to yield injury. Given this complexity, more research is critical to defining these relationships.

In summary, this study demonstrated EXE exacerbated exocrine pancreatic injury in mice on a high fat diet. Morphological changes in the pancreas were time- and dose-dependent upon EXE exposure. Acute pancreatitis and/or pre-cancerous lesions were not seen. Gene expression profiles were indicative of increased stem cell survival and fatty acid oxidation in EXE-treated mice. Increased oxidative stress could be a mechanism for EXE enhanced exocrine pancreatic injury. While the relevance of these findings to human disease remains undefined, the potential for EXE to cause and/or exacerbate pancreatic injury was identified in a HFD mouse model.

## Supporting Information

Table S1 **Mouse weight response to high fat diet and exenatide treatment.**
(DOC)

Table S2 **Serum chemistry changes related to high fat diet and exenatide treatment.**
(DOC)

Table S3 **Exenatide treatment affects serum trypsin and amylase levels.**
(DOC)

Excel Workbook S1 **All individual animal data.**
(XLS)

PDF S1 **IPA Analysis 3 µg Exenatide_vs_control.**
(PDF)

PDF S2 **IPA Analysis 10 µg Exenatide_vs_control.**
(PDF)

PDF S3 **IPA Analysis 30 µg Exenatide_vs_control.**
(PDF)
