# Supplementary material for: Extended Exenatide Administration Enhances Lipid Metabolism and Exacerbates Pancreatic Injury in Mice on a High Fat, High Carbohydrate Diet
Source: PLoS One. 2014 Oct 7;9(10):e109477. doi: 10.1371/journal.pone.0109477 (PMC4188617; doi:10.1371/journal.pone.0109477)
Supplement: Table S1 — Mouse weight response to high fat diet and exenatide treatment. (DOC) [file pone.0109477.s001.doc]

**Table S**1: Mouse weight response to high fat diet and exenatide treatment

| Tx (μg/kg) | 3 week  cohort | 6 week  cohort | 12 week  cohort |
| --- | --- | --- | --- |
| Initial Mean Cohort Weights (Prior to Diet or Treatment) | | | |
| 0 | 29.8 ± 0.5 | 27.6 ± 0.4 | 24.9 ± 0.3 |
| 3 | 30.2 ± 0.6 | 27.3 ± 0.4 | 25.1 ± 0.3 |
| 10 | 28.8 ± 0.8 | 27.9 ± 0.4 | 24.9 ± 0.3 |
| 30 | 29.1 ± 0.5 | 26.2 ± 0.3 | 24.5 ± 0.3 |
| Terminal Mean Cohort Weights (Following Diet and Treatment) | | | |
| 0 | 35.3 ± 1.0 | 39.0 ± 1.1 | 40.6 ± 1.0 |
| 3 | 33.8 ± 1.1 | 37.3 ± 1.0 | 39.2 ± 1.0 |
| 10 | 32.7 ± 1.1 | 36.3 ± 1.0 | 38.0 ± 1.0 |
| 30 | 32.3 ± 1.0* | 32.5 ± 1.0* | 36.8 ± 1.0* |

Tx = treatment; μg/kg = micrograms EXE per kilogram body weight;

* indicates significant difference (p < 0.05) from control; cohort designations

based on weeks of daily EXE treatment.
