# Supplementary material for: Extended Exenatide Administration Enhances Lipid Metabolism and Exacerbates Pancreatic Injury in Mice on a High Fat, High Carbohydrate Diet
Source: PLoS One. 2014 Oct 7;9(10):e109477. doi: 10.1371/journal.pone.0109477 (PMC4188617; doi:10.1371/journal.pone.0109477)
Supplement: Table S2 — Serum chemistry changes related to high fat diet and exenatide treatment. (DOC) [file pone.0109477.s002.doc]

**Table S**2: Serum chemistry changes related to high fat diet and exenatide treatment

| **Tx**  **(μg/kg)** | **Alanine Aminotransferase (U/ml)** | | | **Total Protein (mg/dl)** | | | **Globulin (mg/dl)** | | |
| --- | --- | --- | --- | --- | --- | --- | --- | --- | --- |
|  | **3 Week** | **6 Week** | **12 Week** | **3 Week** | **6 Week** | **12 Week** | **3 Week** | **6 Week** | **12 Week** |
| **0** | 66.3±15.0 | 151.3±16.3 | 96.2±15.0 | 5.0±0.1 | 5.4±0.1 | 5.3±0.1 | 1.43±0.08 | 1.66±0.09 | 1.94±0.08 |
| **3** | 68.4±16.3 | 86.6±15.0* | 85.5±15.4 | 5.1±0.1 | 5.3±0.1 | 5.3±0.1 | 1.52±0.09 | 1.68±0.08 | 1.73±0.08 |
| **10** | 49.4±15.8* | 97.4±15.0* | 55.7±15.8* | 5.0±0.1 | 5.1±0.1 | 5.3±0.1 | 1.55±0.08 | 1.56±0.08 | 1.63±0.08 |
| **30** | 43.2±15.4* | 78.7±15.0* | 46.5±15.0* | 5.1±0.1 | 5.0±0.1* | 5.0±0.1* | 1.54±0.08 | 1.42±0.08* | 1.54±0.08* |

Tx = treatment; μg/kg = micrograms EXE per kilogram body weight; U/ml = units per milliliter; mg/dl = milligrams per deciliter; * indicates significant difference (p < 0.05) from control; Week designations based on weeks of daily EXE treatment.
