# Supplementary material for: Extended Exenatide Administration Enhances Lipid Metabolism and Exacerbates Pancreatic Injury in Mice on a High Fat, High Carbohydrate Diet
Source: PLoS One. 2014 Oct 7;9(10):e109477. doi: 10.1371/journal.pone.0109477 (PMC4188617; doi:10.1371/journal.pone.0109477)
Supplement: Table S3 — Exenatide treatment affects serum trypsin and amylase levels. (DOC) [file pone.0109477.s003.doc]

**Table S3: Exenatide treatment a**ffects serum trypsin and amylase levels

| Tx (μg/kg) | Serum Trypsin (U/ml) | | | Serum Amylase (U/ml) | | |
| --- | --- | --- | --- | --- | --- | --- |
|  | **3 Week** | **6 Week** | **12 Week** | **3 Week** | **6 Week** | **12 Week** |
| 0 | 6.7±0.1 | 8.5±0.3 | 7.9±0.3 | 11965±873 | 10546±637 | 5372±357 |
| 3 | 7.0±0.2 | 8.3±0.3 | 8.0±0.2 | 13886±1097 | 10615±643 | 6530±354 |
| 10 | 6.6±0.1 | 7.0±0.2* | 7.6±0.1 | 12738±1744 | 11781±1156 | 8801±945* |
| 30 | 7.0±0.2 | 7.2±0.2* | 7.1±0.2* | 12525±788 | 13634±840* | 9540±1325* |

Tx = treatment; μg/kg = micrograms EXE per kilogram body weight; U/ml = units per milliliter; * indicates significant difference (p < 0.05) from control; Week designations based on weeks of daily EXE treatment
