# Supplementary material for: Extended Exenatide Administration Enhances Lipid Metabolism and Exacerbates Pancreatic Injury in Mice on a High Fat, High Carbohydrate Diet
Source: PLoS One. 2014 Oct 7;9(10):e109477. doi: 10.1371/journal.pone.0109477 (PMC4188617; doi:10.1371/journal.pone.0109477)
Supplement: PDF S1 — IPA Analysis 3 µg Exenatide_vs_control. (PDF) [file pone.0109477.s005.pdf]

Analysis Name: 3µgExenatide_vs_control
Analysis Creation Date: 2013-12-06
Build version: 261899
Content version: 17199142 (Release Date: 2013-09-17)

Analysis settings

View

Reference set: Mouse Genome 430 2.0 Array

Relationship to include: Direct and Indirect

Includes Endogenous Chemicals

Optional Analyses: My Pathways My List

Filter Summary:

Consider only relationships where

(confidence = Experimentally Observed) AND

(data sources = An Open Access Database of Genome-wide Association Results OR BIND OR BIOGRID OR Catalogue Of Somatic Mutations In Cancer (COSMIC) OR Chemical Carcinogenesis Research Information System (CCRIS) OR ClinicalTrials.gov OR ClinVar OR Cognia OR DIP OR DrugBank OR Gene Ontology (GO) OR GVK Biosciences OR Hazardous Substances Data Bank (HSDB) OR HumanCyc OR Ingenuity Expert Findings OR Ingenuity ExpertAssist Findings OR INTACT OR Interactome studies OR MINT OR MIPS OR miRBase OR miRecords OR Mouse Genome Database (MGD) OR Obesity Gene Map Database OR Online Mendelian Inheritance in Man (OMIM) OR TarBase OR TargetScan Human)

Cutoff:

Fold Change = 1.300

p-value = 5.00E-02

(c) 2000-2014 Ingenuity Systems, Inc. All rights reserved.

Top Networks

ID	Associated Network Functions	Score
1	Energy Production, Lipid Metabolism, Small Molecule Biochemistry	44
2	Behavior, Nervous System Development and Function, DNA Replication, Recombination, and Repair	44
3	Organ Morphology, Tissue Development, Gastrointestinal Disease	40
4	Cancer, Hematological Disease, Reproductive System Disease	40
5	Lipid Metabolism, Small Molecule Biochemistry, Connective Tissue Disorders	36

(c) 2000-2014 Ingenuity Systems, Inc. All rights reserved.

INGENUITY® SYSTEMS

Top Diseases and Bio Functions

Diseases and Disorders

Name	p-value	# Molecules
Connective Tissue Disorders	1.42E-05 - 1.69E-02	12
Metabolic Disease	1.42E-05 - 1.95E-02	67
Gastrointestinal Disease	2.72E-05 - 2.81E-02	193
Inflammatory Disease	2.72E-05 - 2.80E-02	20
Neurological Disease	6.42E-05 - 1.29E-02	78

Molecular and Cellular Functions

Name	p-value	# Molecules
Cell Death and Survival	7.77E-05 - 2.85E-02	186
Cell Morphology	7.51E-04 - 2.58E-02	96
Cell-To-Cell Signaling and Interaction	7.51E-04 - 2.69E-02	32
Lipid Metabolism	9.54E-04 - 2.69E-02	67
Nucleic Acid Metabolism	9.54E-04 - 2.58E-02	14

Physiological System Development and Function

Name	p-value	# Molecules
Connective Tissue Development and Function	4.00E-04 - 2.79E-02	33
Tissue Development	4.00E-04 - 2.58E-02	62
Organismal Survival	4.08E-04 - 2.50E-02	143
Digestive System Development and Function	5.84E-04 - 2.80E-02	60

(c) 2000-2014 Ingenuity Systems, Inc. All rights reserved.

INGENUITY® SYSTEMS

Hepatic System Development and Function	5.84E-04 - 2.80E-02	34

(c) 2000-2014 Ingenuity Systems, Inc. All rights reserved.

Top Canonical Pathways

Name	p-value	Ratio
Fatty Acid β-oxidation I	7.44E-05	7/45 (0.156)
Caveolar-mediated Endocytosis Signaling	2.01E-04	10/85 (0.118)
Virus Entry via Endocytic Pathways	3.11E-04	11/101 (0.109)
RAR Activation	3.58E-03	14/191 (0.073)
Phenylethylamine Degradation I	4.08E-03	2/11 (0.182)

Top Molecules

Fold Change up-regulated

Molecules	Exp. Value	Exp. Chart
ZBTB16	↑9.273	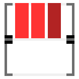
Acot1	↑5.096	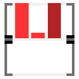
UPP2	↑3.730	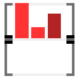
NR1D2	↑3.609	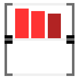
FAM107A	↑3.259	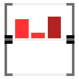
ECH1	↑3.181	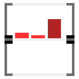

(c) 2000-2014 Ingenuity Systems, Inc. All rights reserved.

RGN	↑2.304	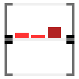
SPHK1	↑2.229	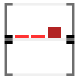
BMP4	↑2.212	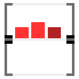
CMBL	↑2.184	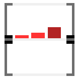

Fold Change down-regulated

Molecules	Exp. Value	Exp. Chart
GADD45G	↓-3.611	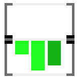
ELOVL6	↓-3.231	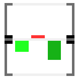
HSPA8	↓-3.108	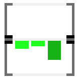
SOAT1	↓-2.811	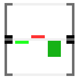
CADM3	↓-2.573	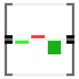
CRY1	↓-2.535	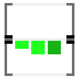
MT1E	↓-2.431	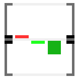
ILDR2	↓-2.392	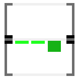
SGK1	↓-2.384	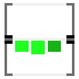
P2RY6	↓-2.360	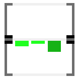

(c) 2000-2014 Ingenuity Systems, Inc. All rights reserved.

INGENUITY® SYSTEMS

Top Upstream Regulators

Upstream Regulator	p-value of overlap	Predicted Activation State
ACOX1	3.44E-06	
EHHADH	8.70E-06	Inhibited
beta-estradiol	1.13E-05	Inhibited
ciprofibrate	1.38E-05	Activated
POR	1.47E-05	

(c) 2000-2014 Ingenuity Systems, Inc. All rights reserved.

INGENUITY SYSTEMS

Top My Lists

Name	p-value	Ratio
My List angiogenesis 769	3.07E-01	30/1023 (0.029)

Top My Pathways

Name	p-value	Ratio

Top Tox Lists

Name	p-value	Ratio
Fatty Acid Metabolism	2.81E-05	14/118 (0.119)
Pro-Apoptosis	3.02E-03	6/42 (0.143)
RAR Activation	3.99E-03	14/176 (0.08)
p53 Signaling	4.63E-02	7/95 (0.074)
Liver Necrosis/Cell Death	5E-02	15/262 (0.057)

(c) 2000-2014 Ingenuity Systems, Inc. All rights reserved.

INGENUITY® SYSTEMS

Top Tox Functions

Assays: Clinical Chemistry and Hematology

Name	p-value	# Molecules
Increased Levels of Red Blood Cells	6.86E-03 - 6.86E-03	9
Decreased Levels of Albumin	3.74E-02 - 2.90E-01	2
Increased Levels of AST	3.67E-01 - 3.67E-01	1
Increased Levels of Blood Urea Nitrogen	4.13E-01 - 4.13E-01	1
Increased Levels of Potassium	4.35E-01 - 4.35E-01	1

Cardiotoxicity

Name	p-value	# Molecules
Cardiac Inflammation	2.39E-02 - 4.57E-01	5
Cardiac Degeneration	3.36E-02 - 3.36E-02	2
Cardiac Arrythmia	3.74E-02 - 1.00E00	5
Cardiac Fibrosis	3.74E-02 - 5.84E-01	9
Cardiac Proliferation	3.74E-02 - 4.60E-01	4

Hepatotoxicity

Name	p-value	# Molecules
Liver Steatosis	4.87E-04 - 1.24E-01	21
Liver Regeneration	5.84E-04 - 1.08E-01	9
Liver Inflammation/Hepatitis	1.68E-03 - 5.17E-01	13
Hepatocellular Carcinoma	1.16E-02 - 3.67E-01	25

(c) 2000-2014 Ingenuity Systems, Inc. All rights reserved.

INGENUITY® SYSTEMS

Liver Hyperplasia/Hyperproliferation	1.16E-02 - 4.77E-01	29

Nephrotoxicity

Name	p-value	# Molecules
Renal Necrosis/Cell Death	5.12E-03 - 1.00E00	19
Glomerular Injury	3.74E-02 - 5.40E-01	12
Nephrosis	3.74E-02 - 3.39E-01	3
Renal Enlargement	3.74E-02 - 1.08E-01	1
Renal Inflammation	3.74E-02 - 1.00E00	8

(c) 2000-2014 Ingenuity Systems, Inc. All rights reserved.
