# Supplementary material for: Extended Exenatide Administration Enhances Lipid Metabolism and Exacerbates Pancreatic Injury in Mice on a High Fat, High Carbohydrate Diet
Source: PLoS One. 2014 Oct 7;9(10):e109477. doi: 10.1371/journal.pone.0109477 (PMC4188617; doi:10.1371/journal.pone.0109477)
Supplement: PDF S2 — IPA Analysis 10 µg Exenatide_vs_control. (PDF) [file pone.0109477.s006.pdf]

Analysis Name: 10µgExenatide_vs_control
Analysis Creation Date: 2013-12-06
Build version: 261899
Content version: 17199142 (Release Date: 2013-09-17)

Analysis settings

View
Reference set: Mouse Genome 430 2.0 Array
Relationship to include: Direct and Indirect
Includes Endogenous Chemicals
Optional Analyses: My Pathways My List

Filter Summary:
Consider only relationships where
(confidence = Experimentally Observed) AND
(data sources = An Open Access Database of Genome-wide Association Results OR BIND OR BIOGRID OR Catalogue Of Somatic Mutations In Cancer (COSMIC) OR Chemical Carcinogenesis Research Information System (CCRIS) OR ClinicalTrials.gov OR ClinVar OR Cognia OR DIP OR DrugBank OR Gene Ontology (GO) OR GVK Biosciences OR Hazardous Substances Data Bank (HSDB) OR HumanCyc OR Ingenuity Expert Findings OR Ingenuity ExpertAssist Findings OR INTACT OR Interactome studies OR MINT OR MIPS OR miRBase OR miRecords OR Mouse Genome Database (MGD) OR Obesity Gene Map Database OR Online Mendelian Inheritance in Man (OMIM) OR TarBase OR TargetScan Human)
Cutoff:
Fold Change = 1.300
p-value = 5.00E-02

(c) 2000-2014 Ingenuity Systems, Inc. All rights reserved.

Top Networks

ID	Associated Network Functions	Score
1	Lipid Metabolism, Small Molecule Biochemistry, Molecular Transport	40
2	Post-Translational Modification, Cell Morphology, Cell Death and Survival	35
3	Cancer, Gastrointestinal Disease, Hepatic System Disease	30
4	Cell Death and Survival, Humoral Immune Response, Protein Synthesis	28
5	Lipid Metabolism, Molecular Transport, Nucleic Acid Metabolism	28

(c) 2000-2014 Ingenuity Systems, Inc. All rights reserved.

INGENUITY® SYSTEMS

Top Diseases and Bio Functions

Diseases and Disorders

Name	p-value	# Molecules
Cancer	1.51E-04 - 2.45E-02	112
Renal and Urological Disease	2.30E-03 - 2.45E-02	9
Endocrine System Disorders	5.83E-03 - 2.45E-02	7
Gastrointestinal Disease	5.83E-03 - 2.45E-02	15
Metabolic Disease	5.83E-03 - 1.23E-02	8

Molecular and Cellular Functions

Name	p-value	# Molecules
Lipid Metabolism	1.84E-06 - 2.45E-02	10
Small Molecule Biochemistry	1.84E-06 - 2.45E-02	13
Cell Death and Survival	3.69E-06 - 2.45E-02	51
Gene Expression	2.01E-05 - 1.23E-02	58
Cellular Development	1.25E-04 - 2.45E-02	51

Physiological System Development and Function

Name	p-value	# Molecules
Hematological System Development and Function	1.05E-04 - 2.45E-02	44
Embryonic Development	1.51E-04 - 2.45E-02	25
Tissue Development	1.51E-04 - 2.45E-02	31
Tumor Morphology	2.83E-04 - 2.45E-02	8

(c) 2000-2014 Ingenuity Systems, Inc. All rights reserved.

Hematopoiesis 9.85E-04 - 2.45E-02 20

(c) 2000-2014 Ingenuity Systems, Inc. All rights reserved.

Top Canonical Pathways

Name	p-value	Ratio
Neuregulin Signaling	3.6E-03	5/104 (0.048)
Thrombopoietin Signaling	4.37E-03	4/64 (0.062)
HGF Signaling	6.66E-03	5/111 (0.045)
Angiopoietin Signaling	7.98E-03	4/75 (0.053)
Erythropoietin Signaling	8.43E-03	4/79 (0.051)

Top Molecules

Fold Change up-regulated

Molecules	Exp. Value	Exp. Chart
ZBTB16	↑5.924	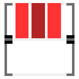
NR1D1	↑5.520	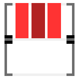
NR1D2	↑4.088	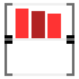
DBP	↑3.379	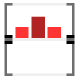
PER3	↑3.322	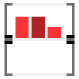
BMP4	↑2.723	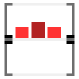

(c) 2000-2014 Ingenuity Systems, Inc. All rights reserved.

INGENUITY® SYSTEMS

TEF	↑2.382	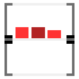
SPHK2	↑2.363	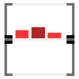
C1orf21	↑2.191	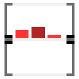
UPP2	↑2.126	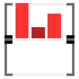

Fold Change down-regulated

Molecules	Exp. Value	Exp. Chart
GADD45G	↓-4.257	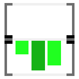
RORC	↓-4.077	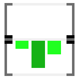
SLC19A2	↓-2.927	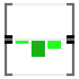
SESN1	↓-2.743	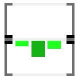
MYCN	↓-2.646	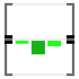
STX1A	↓-2.625	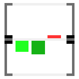
SGK1	↓-2.548	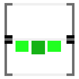
CRY1	↓-2.476	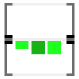
ARNTL	↓-2.434	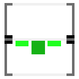
NFIL3	↓-2.382	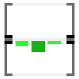

(c) 2000-2014 Ingenuity Systems, Inc. All rights reserved.

Top Upstream Regulators

Upstream Regulator	p-value of overlap	Predicted Activation State
GLI2	6.17E-06	
ZMIZ1	1.87E-05	
progesterone	6.53E-05	
CDK9	1.72E-04	
INHA	2.52E-04	

(c) 2000-2014 Ingenuity Systems, Inc. All rights reserved.

Top My Lists

Name	p-value	Ratio
My List angiogenesis 769	2.83E-01	11/1023 (0.011)

Top My Pathways

Name	p-value	Ratio

Top Tox Lists

Name	p-value	Ratio
Mechanism of Gene Regulation by Peroxisome Proliferators via PPARα	2.89E-02	4/95 (0.042)
VDR/RXR Activation	6.54E-02	3/78 (0.038)
Renal Inorganic Phosphate Homeostasis (Mouse)	7.18E-02	1/6 (0.167)
Vasopressin-induced Genes in Inner Medullary Renal Collecting Duct Cells (Rat)	8.32E-02	1/8 (0.125)
p53 Signaling	9.49E-02	3/95 (0.032)

(c) 2000-2014 Ingenuity Systems, Inc. All rights reserved.

INGENUITY® SYSTEMS

Top Tox Functions

Assays: Clinical Chemistry and Hematology

Name	p-value	# Molecules
Increased Levels of Red Blood Cells	5.32E-03 - 5.32E-03	5
Increased Levels of Alkaline Phosphatase	2.00E-01 - 2.00E-01	2

Cardiotoxicity

Name	p-value	# Molecules
Cardiac Inflammation	6.02E-02 - 3.53E-01	3
Congenital Heart Anomaly	7.18E-02 - 3.53E-01	1
Cardiac Infarction	8.32E-02 - 8.32E-02	1
Cardiac Hypoplasia	1.17E-01 - 1.17E-01	1
Cardiac Fibrosis	1.80E-01 - 1.80E-01	1

Hepatotoxicity

Name	p-value	# Molecules
Biliary Hyperplasia	2.45E-02 - 2.45E-02	1
Liver Hyperplasia/Hyperproliferation	2.45E-02 - 3.20E-01	8
Liver Necrosis/Cell Death	4.01E-02 - 3.11E-01	5
Hepatocellular Carcinoma	1.56E-01 - 2.45E-01	7
Glutathione Depletion In Liver	1.70E-01 - 1.70E-01	1

(c) 2000-2014 Ingenuity Systems, Inc. All rights reserved.

INGENUITY® SYSTEMS

Nephrotoxicity

Name	p-value	# Molecules
Glomerular Injury	1.23E-02 - 1.18E-01	2
Renal Necrosis/Cell Death	1.23E-02 - 1.00E00	5
Nephrosis	1.62E-02 - 6.02E-02	2
Renal Inflammation	2.45E-02 - 3.13E-01	4
Renal Nephritis	2.45E-02 - 3.13E-01	4

(c) 2000-2014 Ingenuity Systems, Inc. All rights reserved.
