# Supplementary material for: Extended Exenatide Administration Enhances Lipid Metabolism and Exacerbates Pancreatic Injury in Mice on a High Fat, High Carbohydrate Diet
Source: PLoS One. 2014 Oct 7;9(10):e109477. doi: 10.1371/journal.pone.0109477 (PMC4188617; doi:10.1371/journal.pone.0109477)
Supplement: PDF S3 — IPA Analysis 30 µg Exenatide_vs_control. (PDF) [file pone.0109477.s007.pdf]

IPA® Interactive pathway analysis of complex 'omics data

INGENUITY® SYSTEMS

Analysis Name: 30µgExenatide_vs_control
Analysis Creation Date: 2013-12-06
Build version: 261899
Content version: 17199142 (Release Date: 2013-09-17)

Analysis settings

View

Reference set: Mouse Genome 430 2.0 Array

Relationship to include: Direct and Indirect

Includes Endogenous Chemicals

Optional Analyses: My Pathways My List

Filter Summary:

Consider only relationships where

(confidence = Experimentally Observed) AND

(data sources = An Open Access Database of Genome-wide Association Results OR BIND OR BIOGRID OR Catalogue Of Somatic Mutations In Cancer (COSMIC) OR Chemical Carcinogenesis Research Information System (CCRIS) OR ClinicalTrials.gov OR ClinVar OR Cognia OR DIP OR DrugBank OR Gene Ontology (GO) OR GVK Biosciences OR Hazardous Substances Data Bank (HSDB) OR HumanCyc OR Ingenuity Expert Findings OR Ingenuity ExpertAssist Findings OR INTACT OR Interactome studies OR MINT OR MIPS OR miRBase OR miRecords OR Mouse Genome Database (MGD) OR Obesity Gene Map Database OR Online Mendelian Inheritance in Man (OMIM) OR TarBase OR TargetScan Human)

Cutoff:

Fold Change = 1.300

p-value = 5.00E-02

(c) 2000-2014 Ingenuity Systems, Inc. All rights reserved.

Top Networks

ID	Associated Network Functions	Score
1	Embryonic Development, Organismal Development, Tissue Development	42
2	Embryonic Development, Tissue Development, Tissue Morphology	40
3	Post-Translational Modification, Protein Degradation, Protein Synthesis	39
4	Hereditary Disorder, Metabolic Disease, Post-Translational Modification	37
5	Molecular Transport, Dermatological Diseases and Conditions, Organ Morphology	35

(c) 2000-2014 Ingenuity Systems, Inc. All rights reserved.

INGENUITY® SYSTEMS

Top Diseases and Bio Functions

Diseases and Disorders

Name	p-value	# Molecules
Endocrine System Disorders	6.86E-06 - 1.88E-02	57
Hematological Disease	6.86E-06 - 2.68E-02	26
Metabolic Disease	6.86E-06 - 2.68E-02	69
Gastrointestinal Disease	1.82E-04 - 2.21E-02	69
Hepatic System Disease	1.82E-04 - 2.21E-02	54

Molecular and Cellular Functions

Name	p-value	# Molecules
Gene Expression	1.73E-05 - 8.05E-03	123
Cell Death and Survival	5.67E-05 - 3.00E-02	189
Cellular Growth and Proliferation	2.01E-04 - 2.96E-02	173
Cellular Development	2.51E-04 - 2.80E-02	119
Lipid Metabolism	3.59E-04 - 2.57E-02	83

Physiological System Development and Function

Name	p-value	# Molecules
Organismal Development	3.52E-05 - 2.80E-02	102
Tissue Morphology	1.33E-04 - 2.77E-02	98
Organismal Survival	1.71E-04 - 2.52E-02	148
Endocrine System Development and Function	2.28E-04 - 1.29E-02	35
Connective Tissue Development and Function	2.51E-04 - 2.76E-02	68

(c) 2000-2014 Ingenuity Systems, Inc. All rights reserved.

Top Canonical Pathways

Name	p-value	Ratio
Ketogenesis	3.36E-04	4/21 (0.19)
NRF2-mediated Oxidative Stress Response	6.24E-04	16/195 (0.082)
Aldosterone Signaling in Epithelial Cells	1.12E-03	14/168 (0.083)
Ketolysis	2.51E-03	3/19 (0.158)
Insulin Receptor Signaling	2.62E-03	12/149 (0.081)

Top Molecules

Fold Change up-regulated

Molecules	Exp. Value	Exp. Chart
ZBTB16	↑19.631	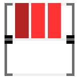
UPP2	↑11.888	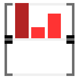
HMGCS2	↑6.779	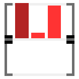
NPTX2	↑5.620	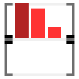
NR1D1	↑4.831	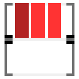
FKBP5	↑4.688	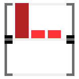

(c) 2000-2014 Ingenuity Systems, Inc. All rights reserved.

INGENUITY® SYSTEMS

Acot1	↑4.658	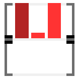
NR1D2	↑4.201	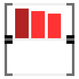
USP2	↑2.974	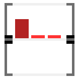
MT1H	↑2.608	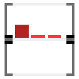

Fold Change down-regulated

Molecules	Exp. Value	Exp. Chart
LDLR	↓-3.851	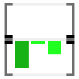
ATAD5	↓-2.904	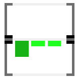
Akr1c14	↓-2.578	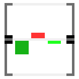
PDE4B	↓-2.511	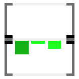
GAS7	↓-2.451	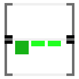
FGF14	↓-2.408	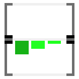
SGK1	↓-2.313	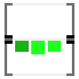
RAB3IL1	↓-2.199	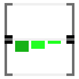
PEG3	↓-2.144	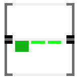
CHIC1	↓-2.066	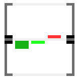

(c) 2000-2014 Ingenuity Systems, Inc. All rights reserved.

Top Upstream Regulators

Upstream Regulator	p-value of overlap	Predicted Activation State
D-glucose	1.11E-05	
FOXO3	2.54E-05	Activated
ARID2	4.96E-05	
PTEN	6.26E-05	
ATP7B	7.20E-05	

(c) 2000-2014 Ingenuity Systems, Inc. All rights reserved.

INGENUITY® SYSTEMS

Top My Lists

Name	p-value	Ratio
My List angiogenesis 769	4.52E-01	28/1023 (0.027)

Top My Pathways

Name	p-value	Ratio

Top Tox Lists

Name	p-value	Ratio
Liver Proliferation	3.07E-03	16/207 (0.077)
NRF2-mediated Oxidative Stress Response	4.96E-03	16/234 (0.068)
Nongenotoxic Hepatocarcinogenicity Biomarker Panel	5.76E-03	4/22 (0.182)
Liver Necrosis/Cell Death	1.31E-02	17/262 (0.065)
PXR/RXR Activation	1.75E-02	6/67 (0.09)

(c) 2000-2014 Ingenuity Systems, Inc. All rights reserved.

Top Tox Functions

Assays: Clinical Chemistry and Hematology

Name	p-value	# Molecules
Increased Levels of Red Blood Cells	6.77E-03 - 6.77E-03	9
Increased Levels of Alkaline Phosphatase	3.84E-02 - 3.84E-02	6
Increased Levels of Hematocrit	2.45E-01 - 2.45E-01	5
Decreased Levels of Albumin	2.90E-01 - 2.90E-01	1
Increased Levels of AST	3.66E-01 - 3.66E-01	1

Cardiotoxicity

Name	p-value	# Molecules
Cardiac Arrythmia	3.73E-02 - 1.00E00	6
Cardiac Fibrosis	3.73E-02 - 5.83E-01	8
Cardiac Inflammation	3.73E-02 - 4.55E-01	4
Cardiac Necrosis/Cell Death	3.73E-02 - 5.67E-01	14
Cardiac Hypertrophy	5.57E-02 - 6.14E-01	17

Hepatotoxicity

Name	p-value	# Molecules
Liver Steatosis	1.82E-04 - 5.84E-02	22
Liver Proliferation	1.45E-03 - 4.17E-01	16
Hepatocellular Carcinoma	5.07E-03 - 2.35E-01	23
Liver Hyperplasia/Hyperproliferation	5.07E-03 - 4.76E-01	29
Liver Inflammation/Hepatitis	1.01E-02 - 1.77E-01	17

(c) 2000-2014 Ingenuity Systems, Inc. All rights reserved.

Nephrotoxicity

Name	p-value	# Molecules
Renal Atrophy	1.10E-03 - 1.10E-03	5
Renal Damage	3.73E-02 - 4.88E-01	9
Renal Inflammation	3.73E-02 - 5.99E-01	11
Renal Nephritis	3.73E-02 - 5.99E-01	11
Renal Tubule Injury	3.73E-02 - 3.40E-01	5

(c) 2000-2014 Ingenuity Systems, Inc. All rights reserved.

INGENUITY® SYSTEMS
